# From Thought to Senses: Assessing the Relationship Between the Generation Effect and Multisensory Facilitation

**DOI:** 10.5334/joc.522

**Published:** 2026-09-28

**Authors:** Michaela Ritchie, Jonathan Wilbiks

**Affiliations:** 1 Department of Psychology, University of New Brunswick Saint John, Canada

**Keywords:** generation effect, multisensory facilitation effect, multisensory learning, verbal memory, cued recall

## Abstract

The current study investigated whether the generation effect, a memory advantage for self-generated verbal information, is enhanced in multisensory conditions. Such a finding would be consistent with the multisensory facilitation effect, a phenomenon wherein multiple sensory inputs may reduce the cognitive load required to process and respond to co-occurring stimuli from multiple senses. Although extensive research has explored the generation effect, the multisensory aspect of this task has been overlooked. In the current study, we aimed to determine if the generation effect is modulated by the multisensory facilitation effect. Multisensory facilitation involves the brain’s ability to efficiently process information from diverse sensory sources simultaneously, enhancing cognitive performance. We employed a 2 (Task Type: generate, read) × 3 (Sensory Modality: auditory, visual, multisensory) factorial design, utilizing word pair lists and a cued recall test. A generation effect emerged for the auditory and audiovisual condition, but not for the visual condition. The hypothesis that the magnitude of the generation effect would be greater for the multisensory encoding condition was not supported. The current study addresses a gap in existing literature by examining the multisensory component of generation tasks and extends the current understanding of multisensory learning by incorporating verbal stimuli as the visual component.

The generation effect refers to the advantage in memory performance for verbal information when it is generated by one’s own mind, rather than passively read (Slamecka & Graf, 1978). The effect is robust, improving both item memory (word-specific) and context memory (information surrounding the word; McCurdy et al., 2020). Although the generation task often inherently involves a multisensory process of interpreting visual stimuli, and vocalizing the text to produce its auditory representation, this aspect of the task has not been explicitly investigated. One specific aim of the current study was to examine whether the generation effect is moderated by multisensory processing.

## Multisensory Facilitation Effect

The ability to efficiently process multisensory information is a capacity finely tuned to accommodate humans’ multisensory way of living (Shams & Seitz, 2008). Complex neural networks throughout the brain support the simultaneous integration and processing of multisensory information (Burton et al., 2002; Falchier et al., 2002; Ghazanfar & Schroeder, 2006; Rockland & Ojima, 2003; Röder et al., 2002). Humans respond and adapt to the multiple sources of information in the environment and reduce cognitive load by pairing congruent stimuli together. Consequently, fewer cognitive resources are needed to process congruent stimuli (Fahey et al., 2018; Shams & Seitz, 2008). Shams and Seitz (2008) define *congruent stimuli* as “[…] the relationship between stimuli that are consistent with the prior experience of the individual or relationships between the senses found in nature” (p.2). By this definition, arbitrary associations between auditory and visual stimuli will not bring about a multisensory facilitation effect. Interestingly, if two stimuli are paired for the purpose of a specific task that are not semantically co-occurring in nature (e.g., an auditory input of a dog barking paired with an image of a lion), integration of these two stimuli during encoding is still possible (Moran et al., 2013). In long-term memory, however, only partial or occasional integration at the subconscious level will occur, leading to deleterious effects of multisensory input of stimuli in such a scenario (Moran et al., 2013; Shams et al., 2011). Congruent pairings are developed within an individual’s semantic network through repeated exposure to those pairings. When associated visual and auditory stimuli are presented together, processing of either stimulus will be facilitated because of a more elaborate representation of the information across the semantic network (Moran et al., 2013; Shams et al., 2011).

Importantly, successful multisensory processing of a single experience is contingent on *temporal binding*, whereby information acquired from each sense be provided within a certain window of time so that the percepts may be unified as a coherent perception (Meredith et al., 1992; Wallace et al., 1996; Wallace & Stevenson, 2014). Failure to adhere to the window could result in the perception of two unintegrated unisensory experiences in lieu of one multisensory percept (Wallace et al., 2004; Stevenson et al., 2012). The precise timing during which stimuli from various modalities are prone to perceptual binding is debated as being as little as 50–100 ms (Slutsky & Recanzone, 2001) to as great as 800 ms (Wallace et al., 2004). Ultimately, the simultaneous presentation of multiple stimuli ensures that auditory and visual stimuli are perceived concordantly, as the temporal window between stimuli provides statistical cues to their relationship (Wallace & Stevenson, 2014).

A notable benefit of multisensory processing is its apparent facilitation effect, wherein unisensory items exhibit enhanced recognition when paired with congruent sensory inputs. Seitz et al. (2006) demonstrated that participants recognized visual stimuli that were paired with concurrent auditory stimuli after approximately 60% fewer training sessions than those who studied visual stimuli in isolation. Multisensory integration may reduce the overall uncertainty in the sensory estimates, leading to more reliable and precise responses (Alais & Burr, 2004). Connections are established through the multisensory learning conditions (Seitz et al., 2006) and any aspect of the multisensory representation can be elicited by merely activating a unisensory component (Friston, 2005; Rao & Ballard, 1999). In support of this, studies have demonstrated a positive relationship between multisensory processing and improved accuracy, precision, and reaction times in various tasks (Alais & Burr, 2004; Ernst & Banks, 2002; Gingras et al., 2009; Kim et al., 2008). Multisensory learning has also been associated with improved detection and localization of stimuli in spatial judgement tasks (Alais & Burr, 2004; Ernst & Banks, 2002; Gingras et al., 2009).

Most research on multisensory facilitation is centered around non-verbal stimuli, but a more recent focus on verbal stimuli has emerged in the literature. Kim (2021) found that the pairing of visual and auditory presentations of the same text improved comprehension compared to when the text was learned by either reading or listening alone. In contrast, Kalyuga (2000) proposed that the learner may incur substantial cognitive load when concurrently processing multiple related sensory sources of information. Consequently, presentation of redundant information across sensory modalities may prove ineffective for learning in some cases (Kalyuga, 2000). Although congruent stimuli from multiple senses may allow for more efficient processing of information, redundant multisensory information is also useful (MacLeod, 2011; Wallace et al., 2004). When the same information is presented to multiple senses at one time (e.g., the visual presentation of a word accompanied by its auditory articulation), the presence of the information from multiple sources increases the probability that the stimuli will be recognized. Redundant multisensory information may enhance the distinctiveness of target stimuli from either sense, a process that has been thought to underlie the generation effect (Kinoshita, 1989). Therefore, the simultaneous presentation of cue and target stimuli in a typical generation task may promote its distinctiveness for later recall (Kinoshita, 1989).

Overall, the literature regarding the advantage of multisensory learning for verbal stimuli is mixed, with many asserting its benefits (Chang, 2009; Kartal & Simsek, 2017; Liu et al., 2019; Mohamed, 2018), and others finding a null (Rogowsky et al., 2016) or negative effect (Diao & Sweller, 2007; Kalyuga, 2000). For example, Diao and Sweller (2007) found that concurrent spoken presentation hindered comprehension and recall of the main ideas of a text compared to the visual-only condition (Diao & Sweller, 2007). More recent research contradicts these findings and indicates that multisensory learning improves comprehension (e.g., Kim, 2021; Liu et al., 2019; Mohamed, 2018). Additionally, students report that multisensory learning facilitates comprehension, shortens the perceived duration of tasks, enhances the appeal of learning, and improves overall sustained attention (Chang, 2009; Kartal and Simsek, 2017). Given the pervasiveness of multisensory learning in academic settings, these indeterminate results emphasize the need for further examination.

## The Generation Effect

Through a similar mechanism to that proposed for the multisensory facilitation effect, the generation effect may emerge by enhancing memory for contextual information related to the generated target (e.g., the cue word; Mulligan, 2004). It has been suggested that the generation effect is more pronounced in overt tasks than in covert tasks (McCurdy et al., 2020), despite the stimuli not being congruent, which warrants further investigation.

Although the contribution of overt (i.e., auditory) verbal generation has not yet been directly examined in the generation effect literature, several studies have included spoken production alongside visual stimulus presentation in the encoding task (e.g., Gardiner & Hampton, 1985; McElroy & Slamecka, 1982; Payne et al., 1986; Slamecka & Graf, 1978). Other studies have implemented covert (i.e., silent) encoding tasks, limiting encoding to a single sensory modality (e.g., McDaniel et al., 1988; Mulligan, 2002; Mulligan et al., 2019). When visual presentation is paired with a vocalized response, the multisensory aspect of the generation task can be characterized as the provision of redundant multisensory information. Despite this, the generation effect is robust, prompting inquiry into whether the multisensory component of the task enhances its effect (increases its magnitude) compared to when an encoding task is unisensory. For instance, there is limited but intriguing meta-analytic evidence from McCurdy et al. (2020) indicating that covert generation tasks (i.e., tasks completed silently) yield slightly smaller effects as compared to overt generation tasks (i.e., tasks completed aloud).

## The Role of Semantic Relatedness of Stimuli

The semantic activation hypothesis suggests that the brain’s ability to recall information is optimized when that information is semantically related, which has been found to underlie the magnitude of the generation effect (Kinoshita, 1989; McCurdy et al., 2020). For instance, meta-analytic findings suggest that a significant generation effect emerged when words or numbers were used as stimuli, but not when nonwords were used (McCurdy et al., 2020). This finding indicates that meaningful information (e.g., words or numbers) may be a necessary condition to obtain a generation effect (McCurdy et al., 2020). The semantic activation theory of the generation effect posits that the act of generation promotes access to semantic information through a number of mechanisms. First, the act of generating a semantically related response to a given cue requires elaborative encoding of the cue word in attempt to create a connection between the new information and existing knowledge (Craik & Lockhart, 1972). The process of generation is therefore more effortful than merely reading, and this increase in cognitive effort may in part contribute to the benefit of generation in memory (Tyler et al., 1979). Non-semantic generation tasks (e.g., letter transposition) lead to smaller benefits (McCurdy et al., 2020) or nonsignificant differences (Kinoshita, 1989) in memory. Free recall is also improved by self-generation during encoding for high-meaningful words but not for low-meaningful words (Kinoshita, 1989; Nairne et al., 1985; Nairne & Widner, 1988). Taken together, these findings suggest that self-generation during encoding enhances the distinctiveness of stimuli when the cue and target stimuli are semantically related, which enhances subsequent recall of the generated information (McCurdy et al., 2020; Kinoshita, 1989).

## Hypotheses

The following hypotheses were derived from a comprehensive examination of relevant literature.

Hypothesis 1 (H1): Based on the robust literature on the generation effect, we predicted a generation effect, as reflected by better cued recall performance for target items that were generated (e.g., Bertsch et al., 2007; McCurdy et al., 2020; Slamecka & Graf, 1978).

Hypothesis 2 (H2): We expected greater recall of target items when they are generated in the multisensory condition compared to when they are generated in the auditory or visual only condition. This hypothesis was formulated in consideration of the literature that multisensory processing improves recognition rates and accuracy of responses, and the limited but intriguing evidence that overt generation tasks yield larger effects than covert tasks (Alais & Burr, 2004; Ernst & Banks, 2002; Gingras et al., 2009; McCurdy et al., 2020).

## Method

### Participants

The current study was approved by the University of New Brunswick’s institutional review board (REB # 2024-012). All aspects of this study, including the recruitment plan, procedure, and analyses, received in principle (Stage I) acceptance through Peer Community in Registered Reports (https://osf.io/3u7eh). Using G*Power (Faul et al., 2009), and specifying “Repeated measures, within factors” per our planned analysis specified below, it was determined that 82 total participants would be required to achieve power of 0.95 to detect a medium effect size (*d* = 0.37), which would be consistent with past work on the phenomenon (see Bertsch et al., 2007, for a review). The effect size included in the power analysis reflects the main effect of task type (read versus generate) we expected to observe. We also tested an interaction effect between task type and sensory modality, which we expected would yield at least a medium effect size, given that both overt and covert generation tasks yield similar effect sizes (see McCurdy et al., 2020, for a review).

To ensure that the order of the tasks was counterbalanced evenly across participants, we recruited for a final sample size of 84 participants. To ascertain the quality of participant data, participants were recruited until we obtained useable data from 84 participants. Thus, 96 participants were tested. Seven participants were excluded from analysis for terminating the study early, one participant was excluded due to failure of the microphone during testing which prevented auditory responses from being recorded, and four participants were removed from analysis because they completed at least one of the tasks incorrectly. Instances of non-compliance with the study’s instructions appeared to be due to a misunderstanding in task instructions, such as repeating the cue word during encoding, instead of generating a target word for the word pairs, despite feedback given after the practice trials. All participants were required to respond to all cue words during the encoding phase, providing either a target word, or indicating that they had no response. This ensured that any missing information in the task is due to lack of knowledge rather than accidental omission. This decision is based on the need to ensure that any missing responses are attributable solely to a lack of knowledge, rather than to an inadvertent oversight. A total of 96 participants were tested, and after the exclusion of 12 participants, our final sample size was achieved (*N* = 84). Participants were only included in the analysis if all tasks were appropriately completed as outlined in the procedure.

Participants were recruited from the researchers’ university population of undergraduate students enrolled in a Psychology course. In compensation for their participation, participants were awarded one bonus credit toward their Psychology course or a $10 gift card. Of the final sample, 59 participants were female, 22 participants were male, and three participants identified as either non-binary (*n* = 1) or transgender (*n* = 2). The mean age of the sample was 21.87 years old (*SD* = 6.26 years), and most of the sample (83.33%) reported English as being their first language. The data were examined for outliers, which were defined as any score that was greater than three standard deviations from the mean and discontinuous from the distribution of scores. Using the mean and standard deviation of the outcome (*M* = 0.71, *SD* = 0.19), the cutoff value was calculated as any score below 0.14 (or above 1.28, which is not possible, given the proportional nature of the data). One observation (0.10) was smaller than the cutoff value but was not discontinuous from the rest of the distribution. As an additional check, the planned analyses were conducted with both inclusion and exclusion of that participant, and the pattern of results did not change, so the observation was retained in the dataset.

### Materials and Procedure

The stimuli used for the current study were 120 word pairs taken from the Nelson free association norms (Nelson et al., 2004; see supplementary materials). All materials have been uploaded to an online repository (https://doi.org/10.17605/OSF.IO/SKQR7). The cue and target words of each word pair are semantically associated (forward associative strength: *M* = 0.23, *SD* = 0.08, range = 0.102–0.397; backward associative strength: 0; letter length: *M* = 6.49, *SD =* 0.16, range = 3–11). Using a fully within-subjects counterbalanced blocked design, some participants were first exposed to the completed word pair lists under the reading conditions, and the other participants first received the incomplete word pair lists—which contained only the cue words and the first letter of each target word—for the generation tasks. The experiment was programmed and administered using PsychoPy, a freely available software for conducting psychological studies (Peirce et al., 2019). All stimuli were presented using this software, in addition to instructions about whether the stimuli would be presented through an auditory, visual, or multisensory modality preceding each condition. Instructions and practice trials were presented at the beginning of the study.

Where relevant, each word was displayed in PsychoPy in Arial black font, with a grey background. Most letters were 25.40 mm in height, except for stemmed letters (e.g., d, b, p). For conditions where a visual display was accompanied by a pre-recorded auditory articulation of the cue word, a pre-recorded articulation of that word accompanied its visual presentation and was synchronized using PsychoPy and presented at 50% volume. A textbox was included on a blank grey screen for visual conditions, where participants were instructed to input their target word response in real time. Auditory responses were recorded with a microphone and saved within the experiment. All participants were tested in a quiet room on a Dell monitor (53.78 × 16.60 cm). Where relevant, the screen was displayed at full brightness. Each word pair had a presentation and response duration of 4 s, such that the participant either received both words (in the reading conditions) or received the cue word and generated the target word (in the generation conditions) in this time frame. In all conditions, each word pair trial will be followed by a blank interstimulus interval (ISI) of 1 s. The display was advanced to the next screen after the allotted 4 s and a 1 s ISI.

#### Auditory Reading Condition

An auditory articulation of the cue word, which was pre-recorded by the researcher, was played via PsychoPy. The auditory articulation of the target word was also played via PsychoPy. A grey screen accompanied the auditory presentations.

#### Visual Reading Condition

Both the cue word and the target word were presented on the screen. Participants read each word of the word pair silently.

#### Multisensory Reading Condition

Both the cue word and the target word were presented and accompanied simultaneously by an auditory articulation of each word which was pre-recorded by the researcher.

#### Auditory Generation Condition

A pre-recordered articulation of the cue word and the first letter of the anticipated target word was played. A grey screen accompanied the auditory presentations. The participant vocalized their response (i.e., the target word), which was recorded in PsychoPy.

#### Visual Generation Condition

The cue word, and the first letter of the anticipated target word, were presented on the screen. Participants generated their response by typing out the target word, which was displayed on the screen.

#### Multisensory Generation Condition

The cue word was presented on the screen and accompanied simultaneously by an auditory articulation of that word. Participants generated their response by typing out the target word, which was displayed on the screen, and was then articulated aloud by the participant.

#### Distraction Task

Following each encoding task, participants were given a number on the screen, and were instructed to continually subtract seven from the given number, and to input each response in PsychoPy, for 30 seconds.

#### Cued Recall Test

A total of six untimed cued recall tests were administered following each distraction phase. One at a time, a cue word was displayed on the screen, in a randomized order. For each cue word that was displayed, the participant was instructed to type in the associated target word, and their response appeared on the monitor. Once they submitted their response, they were advanced to respond to the next cue word after a 1 s ISI.

#### Procedure

Following a fully within-subjects blocked design, participants were exposed to 60 complete word pairs under the reading condition, and 60 incomplete word pairs under the generation condition, and the order in which they were exposed to either task was counterbalanced across participants. After signing their informed consent, they were given explicit instructions in PsychoPy about how each task would be completed. Instructions and examples for each condition were provided to ensure that participants were familiar with the expectations of each type of task. Participants then encoded a total of 120 word pairs, half of which were self-generated. Under each of the six conditions, 20 word pairs were encoded. Which word pairs appeared under each condition was counterbalanced across all participants so that there was equal exposure to each word pair list under each condition, to ensure that any effect observed was attributable to the encoding condition.

Following each of the six encoding conditions, participants engaged in a 30 s distraction task before responding to a cued recall task for the items on the previous trial. The presentation of the cue words in each test was in a randomized order. After all six conditions were completed, participants were given a debriefing form to explain the purpose of the study.

## Results

Prior to data collection, all research questions, hypotheses, and planned analyses were specified as part of a Stage I registered report (see Table S1 in supplementary materials). The data obtained from the current study are available on the Open Science Framework (https://doi.org/10.17605/OSF.IO/SKQR7). To calculate the rate of successful generation across conditions, responses were first classified as omissions when no response was provided during encoding, and as commissions when the response did not match the anticipated target word. The number of correct generation responses was then calculated as the total number of trials (i.e., 20) minus the sum of omission and commission errors.

Successful generation across all conditions was low. On average, participants correctly generated approximately 41% of targets (*M* = 8.17, *SD* = 3.32), indicating a relatively high rate of errorful generation. The rate of successful generation differed significantly across sensory conditions: *F*(2, 166) = 13.34, η_p_^2^ = .14, *p* < .001, and post hoc comparisons indicated that it was significantly lower in the visual condition compared to both other sensory conditions (for pairwise comparisons, see Table 1; commission and omission error rates and mean successful generation rates across sensory conditions are reported in Table 2). The rate of errorful generation across conditions was further explored with a repeated-measures ANOVA, and the omnibus test was significant: *F*(2, 166) = 23.31, η_p_^2^ = .22, *p* < .001. Post hoc comparisons with Bonferroni corrections indicated that a significantly greater number of omission errors were made in the visual condition compared to the auditory condition: *t*(83) = 5.31, *SE* = 0.29, *d* = 0.70, *p* < .001, and compared to the audiovisual condition: *t*(83) = 5.77, *SE* = 0.28, *d* = 0.73, *p* < .001. The number of omission errors did not significantly differ between the audiovisual and auditory conditions: *t*(83) = 0.27, *SE* = 0.23, *d* = 0.03, *p* = .999. The number of commission errors was also compared across sensory encoding conditions. In the current study, any deviation from the anticipated target word was considered an error of commission. The omnibus test was non-significant: *F*(2, 168) = 2.45, η_p_^2^ = .03, *p* = .089, indicating that the number of commission errors did not significantly differ across sensory encoding conditions.

**Table 1:** Pairwise Comparisons of Successful Generation Rates Across Sensory Conditions.


SENSORY MODALITY		MEAN DIFFERENCE	*t*(83)	*SE*	*p*

Visual	Auditory	–1.06	–3.09	0.34	.008

	Audiovisual	–2.06	–4.79	0.43	<.001

Audiovisual	Auditory	1.00	2.39	0.42	.057


*Note*. Mean differences represent Bonferroni-adjusted pairwise comparisons of successful generation rates across sensory conditions. Positive values indicate higher successful generation in the first-listed condition relative to the comparison condition, whereas negative values indicate lower successful generation. SE = standard error.

**Table 2:** Mean Error Rates and Rates of Successful Generation Across Sensory Conditions.


	SENSORY CONDITION	*M*	*SD*

Omission Errors	Auditory	1.81	1.80

	Visual	3.35	2.66

	Audiovisual	1.75	2.01

Commission Errors	Auditory	10.00	2.93

	Visual	9.52	3.82

	Audiovisual	9.06	3.45

Successful Generation	Auditory	8.19	2.75

	Visual	7.13	3.24

	Audiovisual	9.19	3.64


*Note*. Reported values reflect the average number of omission errors and commission errors made in the generation task in each sensory condition, out of 20 trials, as well as the average number of successfully generated targets during encoding across sensory conditions. M = mean; SD = standard deviation.

Given the high rate of errorful generation during encoding, recall accuracy was calculated as the proportion of trials on which participants reproduced their originally generated response to the corresponding cue at test, out of 20 per trial. For read conditions, the outcome (i.e., cued recall accuracy) was calculated as the proportion of correctly recalled words out of 20 per trial. In both cases, responses were scored as correct only when the target word was recalled in association with its corresponding cue; correct targets produced for incorrect cues were not counted as correct. To analyze the effect of encoding modality and sensory modality on the outcome, cued recall performance, the following planned analysis was conducted: A 2 (Task Type: generate, read) × 3 (Sensory Modality: auditory, visual, multisensory), factorial analysis of variance (ANOVA), with task type assessed within subjects, and sensory modality between subjects. Group mean statistics for encoding and sensory conditions are reported in Table 3.

**Table 3:** Group Mean Statistics.


ENCODING TASK	AUDITORY	VISUAL	AUDIOVISUAL

Read	0.66 (0.19)	0.66 (0.20)	0.65 (0.19)

Generate	0.77 (0.17)	0.74 (0.19)	0.78 (0.17)


*Note*. *N* = 84. Mean proportions (and standard deviations) for each group are reported. Encoding task (read, generate) and sensory condition (auditory, visual, audiovisual) were measured within subjects.

H1 specified that a main effect of task type would be observed, with participants performing better in the generate condition compared to targets that were read, as reflected by greater recall for target items that were generated. In support of this hypothesis, there was a significant main effect of encoding: *F*(1, 83) = 29.99, η_p_^2^ = .27, *p* < .001, observed power = 1.00. Overall, generated items were better recalled than read items, *M*_diff_ = 0.11, *p* < .001.

Given that H2 specified a generation effect would emerge across all sensory conditions but would be larger in magnitude for the multisensory condition, an ordinal interaction was assessed (Elias, 2004; Park & Yi, 2024). First, the 2 × 3 factorial ANOVA specified above was conducted to assess whether sensory condition moderated the strength of the relationship between task type (read/generate) and cued recall (outcome of interest). The assumption of sphericity was not met for the main effect of sensory modality, so Greenhouse-Geisser values are reported to correct for the violation. A main effect of sensory modality did not emerge as statistically significant: *F*(1.77, 147.01) = 0.83, η_p_^2^ = .01, *p* = .425, observed power = 0.67, and sensory modality did not significantly interact with task type: *F*(2, 166) = 2.03, η_p_^2^ = .02, *p* = .134, observed power = 0.95. Although it was expected that a generation effect would emerge at all levels of the sensory condition, post hoc tests indicated that the generation effect only emerged for the auditory condition: *M*_diff_ = .11, *t*(83) = 4.71, *SE* = .02, *d* = 0.58, *p* < .001, and the audiovisual condition: *M*_diff_ = .13, *t*(83) = 5.41, *SE* = 0.02, *d* = 0.42, *p* < .001. The generation effect did not reach statistical significance in the visual condition: *M*_diff_ = .08, *t*(83) = 2.95, *SE* = 0.03, *d* = 0.71, *p* = .062. For a visual representation of the effect across sensory conditions, see Figure 1.

**Figure 1:**
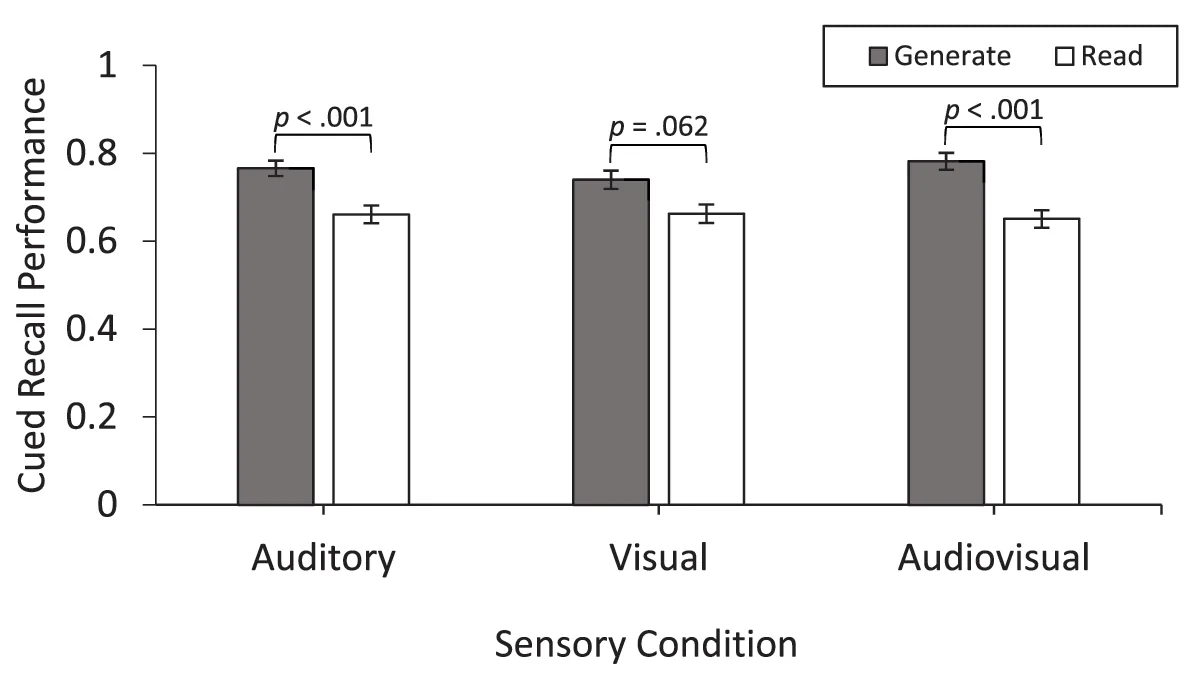
Generation Effect Across Sensory Modalities. *Note*. The size of the generation effect across sensory encoding conditions (auditory, visual, audiovisual), measured as the proportion of items correctly recalled in cued recall test. Error bars represent standard errors of the mean.

We hypothesized a significant interaction between sensory modalities and task type, such that the magnitude of the generation effect was expected to increase in the multisensory condition, but the direction of the effect would not change at any level of sensory modality. To ensure the observed effects were not an artifact of the scale of measurement, a logit transformation was applied onto the dependent variable (Labaronne et al., 2023; Wagenmakers et al., 2012) and the planned analysis was repeated. The main effect of encoding survived the transformation, indicating that it was not an artifact of the original scale of measurement: *F*(1, 83) = 21.77, η_p_^2^ = .21, *p* < .001. The main effect of sensory modality remained non-significant, *F*(2, 166) = 0.05, *p* = .950, as did the interaction term, *F*(2, 166) = 0.65, *p* = .524. A significant generation effect emerged for the auditory condition, *t*(83) = 3.72, *SE* = 0.22, *p* = .005, as well as for the audiovisual condition, *t*(83) = 4.28, *SE* = 0.21, *p* < .001. Consistent with the results obtained from the original proportions, no significant difference in cued recall performance emerged in the visual condition: *t*(83) = 2.28, *SE* = 0.26, *p* = .380. The size of the generation effect was not found to significantly differ as a function of sensory condition (for all comparisons, Bonferroni adjusted *p* = .999). In summary, the same pattern of results was observed for the logit-transformed data (see Table 4).

**Table 4:** Post Hoc Comparisons of Generation Effect Size Across Sensory Conditions.


	SENSORY MODALITY		*M*_diff_	*t*	*SE*	*df*	*p*_bonf_

Original proportions	AV	A	0.03	0.72	0.03	249	.999

	AV	V	0.05	1.53	0.03	249	.380

	A	V	0.03	0.81	0.03	249	.999

Logit-transformed values	AV	A	0.05	0.16	0.33	249	.999

	AV	V	0.30	0.91	0.33	249	.999

	A	V	0.25	0.76	0.33	249	.999


*Note*. Post hoc comparisons of the size of the generation effect, calculated as the mean difference in cued recall performance for generated targets and read targets of the same sensory condition. Mean differences should be interpreted for original proportions. Logit-transformed values are presented to corroborate the original analysis, indicating that the obtained results are not an artifact of the distribution of proportions. A = Auditory sensory condition. V = Visual sensory condition. AV = Audiovisual sensory condition. pbonf = Bonferroni-adjusted p-values for comparing a family of three estimates.

### Exploratory Analyses

Because participants in the generation condition did not consistently produce the intended target items during encoding, multiple scoring approaches were considered to evaluate the robustness of the generation effect. For generation conditions, the primary results reported here are based on the number of targets correctly recalled out of 20 trials, regardless of whether the encoded response matched the anticipated response for encoding. Recall accuracy was therefore measured in reference to the actual encoded response, not the anticipated encoded response. This approach introduces a potential confound, as items that participants successfully generated during encoding may have been selected for generation because those words were already more accessible in memory, which may inflate the generation effect when compared to the pre-selected targets presented in the read conditions. To address this, an alternative scoring approach was considered in which recall accuracy was calculated conditional on successful generation during encoding (i.e., proportion of correctly recalled items out of those correctly generated). Although this method conditionalizes memory performance on successful encoding, it necessarily reduces the denominator for generate trials (but never for read trials) and may therefore inflate estimates of the generation effect.

A third approach that was considered was soring recall as the number of correctly recalled items out of all 20 trials regardless of encoding success. However, this method may underestimate the generation effect by conflating failures of generation with failures of memory. To more directly address this limitation, an additional sample (*N* = 57) was tested in which feedback was provided following each generation attempt. This ensured that all participants were exposed to the intended target items, regardless of initial generation success, thereby equating encoding across conditions. Prior research indicates that an attempt to generate the correct target is sufficient to yield a generation effect, if feedback is given (Clark et al., 2021; Begg et al., 1980; Kornell et al., 2009; Potts & Shanks, 2014; Slamecka & Fevreiski, 1983). The complete pairing was displayed in all reading conditions to equate total exposure time, and feedback was given through the same modality of that sensory condition (e.g., visual feedback was shown for the visual encoding condition). Feedback was displayed for an additional 4 s after the encoding window for the target had elapsed.

In the feedback sample, 72.41% of participants reported English as their first language, majority of participants were female (74.14%) and the average age of the sample was 21.82 years old (*SD* = 5.81). Analyses of this feedback condition revealed a robust main effect of encoding task, *F*(1, 56) = 95.08, η_p_^2^ = .63, *p* < .001, observed power = 1.00, indicating a strong generation effect. There was no significant main effect of sensory modality, *F*(2, 112) = 2.46, η_p_^2^ = .04, *p* = .090, observed power = 0.99, nor a significant interaction between encoding task and sensory modality, *F*(2, 112) = 1.50, η_p_^2^ = .03, *p* = .230, observed power = 0.92. Exploratory simple main effects analyses confirmed that the generation effect was significant across all sensory conditions (in all cases, *p* < .001; see Table S2).

## Discussion

Despite having a vast body of literature to rely on when examining the effect of self-generation on memory under a host of different conditions (for reviews of this literature, see Bertsch et al., 2007; McCurdy et al., 2020), the multisensory component inherent to most generation tasks has been neglected as an area of focus in this research. To help understand the mechanisms underlying the generation effect, we assessed whether the multisensory component of the task would modulate the size the effect. This examination was necessary to elucidate whether generation tasks are exemplary of the multisensory facilitation effect, or whether the generation effect is as pronounced when the multisensory component of generation tasks is absent.

In support of H1, a main generation effect was observed in the current study, which is consistent with prior literature that the benefit is robust across an array of experimental task designs (McCurdy et al., 2020). Interestingly, the significant main effect was driven by significant effects in the audiovisual and auditory encoding tasks, but no significant generation effect emerged for the visual encoding task. Although meta-analytic findings suggest that generating by speaking may yield larger effects than generation tasks that are completed by writing or typing (McCurdy et al., 2020), a plethora of studies demonstrate a significant effect for written generation tasks (for reviews, see Bertsch et al., 2007; McCurdy et al., 2020).

In consideration of transfer-appropriate processing and encoding specificity accounts (Morris et al., 1977; Thomson & Tulving, 1970), the finding of a generation effect in the auditory and audiovisual conditions, but not in the visual condition, may seem surprising. According to these accounts, memory performance is typically enhanced when the operations engaged at retrieval overlap with those engaged during encoding (McDaniel et al., 1988; Morris et al., 1977; Thomson & Tulving, 1970). The cued recall test employed in the current study required typed visual responding from the participants, regardless of the sensory modality in which the information was encoded. Therefore, one might expect the visual generation condition to benefit most from an encoding-retrieval overlap. Interestingly, however, significant generation effects emerged in the auditory and audiovisual conditions, but not in the visual condition. Although the visual generation condition arguably shared the greatest procedural overlap with the retrieval process, the current findings suggest that sensory overlap may not be a substantial predictor of performance. The central tenet of these theoretical accounts concerns the similarity of cognitive operations engaged at encoding and at retrieval, which is not constrained to mere sensory modality overlap. Therefore, it is important to consider how encoding demands may have differed qualitatively across sensory conditions to yield the observed results.

One possibility is that the auditory task facilitated phonological elaboration which enhanced the distinctiveness of the cue-target relationship, and may have also stimulated attention, compared to the silent, visual generation task (MacLeod et al., 2011). Another possibility, discussed in greater detail below, is that the visual generation task introduced greater perceptual task demands associated with typing a response under a time constraint, which may have consequently attenuated any benefit of generation. The proposed explanations are not mutually exclusive and likely interacted to influence the extent to which generation supported later retrieval across sensory conditions. Thus, the present findings do not necessarily contradict principles of transfer-appropriate processing or encoding specificity. Instead, the critical overlap in processes likely concerned cue-target relational processing required for successful cued recall performance (McDaniel et al., 1988). For the reasons outlined above, cue-target relational processing may have been encoded more distinctively in the sensory conditions that included an auditory generation component.

As proposed at Stage I of the current study, a null effect for the visual generation task may most likely indicate that the task at hand did not provide sufficient opportunity to encode the target material in the learning trials for the generation tasks. When presented with a cue word, the learner must mentally search their semantic network to generate a suitable target, given the rule and target letter provided by the researcher. This reflects the covert process of generation (i.e., thinking about the generated response). Given that overt generation mechanisms were manipulated in the current study, the covert process must then necessarily be followed by an overt generation task. The overt tasks were manipulated as (a) typing the target in the visual sensory condition; (b) saying it aloud in the auditory sensory condition, or (c) typing and then saying the target aloud in the audiovisual sensory condition. From the null effect observed only in the visual condition, where responses are only typed, we speculate that participants experienced an insufficient amount of time to overtly generate their responses. Typing out a word is more time consuming than merely saying it aloud, and perceived time pressure in the visual condition may have adversely impacted encoding in this condition. In previous studies, responses to a visual learning task were handwritten, and in the current study, responses were typed. To type a response, participants would have had to orient themselves to the layout of the keys and possibly gauge their spelling by shifting attention from the keyboard to the screen. Although typing was required for audiovisual generation as well, the typing task preceded the auditory articulation of the same generated response. Therefore, although participants may not have had sufficient time to adequately process the cue-target relationship before the next trial in the visual-only condition (Burns, 1992), the time pressure may have played less of a contributing role in an audiovisual condition.

In the audiovisual condition, initially determined responses were given additional opportunity for processing. That is, after a response was typed in the audiovisual condition, that same target was also said aloud by the participant. The addition of the auditory component to the visual task may have served as an opportunity for the learner to reinforce the encoding of the target that was initially generated by typing, which may have mitigated a perceived time pressure across trials. If perceived time pressure negatively affected performance in the visual-only condition, this should be reflected in a greater number of omission errors, suggesting that participants lacked sufficient time to generate, record, and adequately encode responses before the next trial. This claim is supported by the results of the ANOVA comparing the number of omission errors (i.e., no response given) in the visual condition compared to the other sensory conditions. Additionally, no significant difference should emerge for the auditory and audiovisual tasks, if only the visual condition was particularly susceptible to a perceived time pressure, which was also supported by the results of the ANOVA.

Commission errors may also reflect insufficient time for response preparation, as partial responses (e.g., *guil* when the anticipated target was *guilty*) are not strictly omission errors, but are nonetheless indicative of time pressure. Importantly, commission errors also reflect complete, but incorrect targets, (e.g., *later* when the anticipated target was *leave*), meaning that the rate of commission errors across conditions does not provide direct evidence of a time pressure. Importantly, it is not possible to objectively distinguish commission errors arising from incomplete responses from those reflecting fully formed but incorrect responses. For the anticipated target *clothes*, it cannot be determined whether *cloth* was generated as a truncated response due to perceived time pressure or was instead intentionally selected by the participant as a suitable response. As another example, commission errors may reflect the selection of shorter or easier-to-type responses under perceived time pressure (e.g., *silly* instead of the anticipated target *stupid*), but this cannot be determined from the data. Accordingly, all commission errors were treated uniformly; however, they should not be interpreted as direct evidence of perceived time pressure. The results indicated that the number of commission errors did not significantly differ across sensory encoding conditions.

Given the robustness of the generation effect reported in the literature across a host of experimental designs (Bertsch et al., 2007; McCurdy et al., 2020), it is unlikely that a non-significant generation effect is attributable to an absence of an actual benefit. Rather, it is more reasonable that the structure of the task itself prevented the typical generation effect from emerging. Although the time constraint in the current study was selected based on prior studies (e.g., Gardiner et al., 1988; Johns & Swanson, 1988; Kinoshita, 1989; Mulligan et al., 2019; Slamecka & Fevreiski, 1983; Slamecka & Graf, 1978), it may have been inadequate in the current sample, particularly when considering that participants had to type responses within the allotted time. To help corroborate this claim, data were collected on a separate sample where feedback was given following generation attempts. Although those analyses were not planned, the results indicated a significant generation effect in all sensory conditions. This may support the claim that in the planned methodological approach, participants experienced an insufficient opportunity to fully encode their generated response. The provision of feedback may have provided an opportunity to process and consolidate the target before attention was redirected to the next trial, thereby eliminating a perceived time pressure and improving performance. Additional work on the role of feedback would be needed to ascertain this possibility.

The rate of successful generation in the current study was relatively low, which may suggest that the present materials did not constrain responses as tightly as intended. Many other generation effect paradigms employed highly constrained cue-target relations derived, and reported higher success rates (e.g., Gardiner et al., 1988; McElroy & Slamecka, 1982; Mulligan, 2002; Mulligan et al., 2019; Slamecka & Graf, 1978). One possible explanation is that Nelson’s free association norms (Nelson et al., 2004), which were used to select the cue-target pairs, may no longer accurately reflect associative responses within the current population. Additionally, only the first letter of the anticipated target was provided, which may have been insufficient to guide participants toward a single anticipated response. Given that cue-target pairs were semantically related and participants were instructed to generate any associated word beginning with the specified letter, variability in responses is not entirely unexpected, as individuals differ in their associative networks based on prior experience (Agustín-Llach, 2023; Leger et al., 2024; Morais et al., 2013).

The total number of exposures (i.e., through auditory and visual media) was equated in the audiovisual reading condition, so a significant generation effect in the audiovisual condition is not attributable to total number of exposures to the target. The findings of the current study suggest that compared to a visual generation task, the addition of an auditory component may benefit the processing of the generated response in memory for later retrieval, particularly when the encoding window is brief. This would be consistent with the notion that the multisensory presentation of redundant information increases the probability that the stimulus will be recognized and responded to, because of its enhanced distinctiveness (MacLeod, 2011; Wallace et al., 2004). Thus, although the current results do not support the hypothesis that multisensory generation yields superior performance to unisensory generation, they do indicate that multisensory responding may, in some cases, aid processing of a cue-target relationship, particularly when the encoding interval is brief. More research is needed to ascertain this possibility.

Overall, the results do not offer uniform support for H2, which proposed that the magnitude of the generation effect would be larger for the audiovisual condition compared to either unisensory condition. The interaction between sensory modality and encoding task was non-significant, which may indicate that the effects of overt generation tasks (i.e., reading the cue word and generating the target aloud) that have been reported in the past (e.g., McCurdy et al., 2020), may be better explicated by chance error variance, or other factors. For example, multisensory generation may provide the learner with an opportunity to consolidate their covertly generated response to a greater extent than in unisensory conditions, as proposed above. Although this may yield a significant benefit under perceived time pressure, when compared to a visual task, it does not provide evidence that multisensory generation is inherently superior to unisensory generation. For example, the same benefit was not observed for the auditory comparison, suggesting that the addition of a visual component to an auditory generation task did not significantly contribute to performance. Thus, the observed pattern of results across sensory and encoding conditions might suggest that the addition of an overt articulation of a generated target is beneficial for encoding to reinforce responses under perceived time pressure, but this conclusion is speculative, given that no measure of perceived time pressure was collected. Ultimately, lack of support for H2 indicates that the multisensory component of the generation task did not uniformly improve retention of the target items compared to unisensory generation tasks.

Extant literature on the multisensory facilitation effect is mixed. Some findings suggest that multisensory processing can enhance recognition of target stimuli and improve accuracy and precision of responses (Alais & Burr, 2004; Ernst & Banks, 2002, Gingras et al., 2009). Beyond that, multisensory learning has been associated with improved comprehension outcomes (Kim, 2021; Liu et al., 2019; Mohamed, 2018), although others have found null or deleterious effects on comprehension (Diao & Sweller, 2007; Kalyuga, 2000; Rogowsky et al., 2016). In the current study, we sought to test whether the multisensory facilitation effect emerged for redundant presentations of verbal stimuli (i.e., word pairs), and further, to evaluate whether such an effect would be enhanced for generated information. First, we tested the proposal that when the same information is presented to multiple senses during learning, the probability of recognition of that stimulus is enhanced (MacLeod, 2011). Given that the effect of sensory modality was not significant, our results do not offer uniform support for this claim. Additionally, given that generating during learning is also believed to increase the probability of target recognition (McDaniel et al., 1988; Slamecka & Graf, 1978), we tested whether generated targets would be increasingly advantaged in recognition when learned under a multisensory condition, compared to passively encoded targets. Overall, our results do not offer uniform support for either claim.

### Limitations and Future Directions

The most surprising finding from the current study was a null generation effect in the visual learning condition, which is inconsistent with a large body of literature exhibiting a generation effect for written tasks (McCurdy et al., 2020). As discussed, we suggest that participants may have perceived a time pressure during the visual generation task that precluded the typical effect from being observed. To evaluate this, measures of perceived time pressure under different encoding tasks could be helpful. In the current study, exploratory analyses from a separate sample in which feedback was provided after each generation attempt offer informative evidence regarding this issue. The provision of feedback allowed encoding to be verified across sensory conditions, and under these conditions a robust generation effect emerged in the visual condition. These findings suggest that the absence of a generation effect in the visual condition may reflect a procedural constraint related to encoding demands, rather than a genuine absence of a memory benefit. Although the exploratory analysis was conducted on a smaller sample, the obtained results offer evidence that the null effect in the visual condition of the primary analysis reflected a procedural constraint rather than a true absence of an effect. In future work, it would be useful to increase the exposure time to each word pair under all conditions. Although previous studies of the generation effect have employed a 4s interval (e.g., Johns & Swanson, 1988; Slamecka & Fevreiski, 1983), a longer encoding interval may be more appropriate under the current paradigm where the participant is required to adapt to different task demands across encoding and sensory conditions. Increasing the encoding interval in future work may alleviate perceived demands and yield a typical generation effect.

The multisensory facilitation effect has been primarily examined using non-verbal stimuli, and the limited findings on the effect of language comprehension have yielded mixed results. Overall, findings from the current suggest that multisensory learning does not uniformly improve cued recall relative to unisensory learning, across both passive (listening, reading) and active (vocalized or typed generation) learning conditions.

## Data Availability

The data for this project are publicly available on the Open Science Framework (https://doi.org/10.17605/OSF.IO/SKQR7).
